# Redistribution of Actin during Assembly and Reassembly of the Contractile Ring in Grasshopper Spermatocytes

**DOI:** 10.1371/journal.pone.0004892

**Published:** 2009-03-16

**Authors:** G. Bradley Alsop, Wei Chen, Margit Foss, Kuo-Fu Tseng, Dahong Zhang

**Affiliations:** 1 Department of Zoology, Oregon State University, Corvallis, Oregon, United States of America; 2 Molecular and Cellular Biology Program, Oregon State University, Corvallis, Oregon, United States of America; 3 Center for Genome Research and Biocomputing (CGRB), Oregon State University, Corvallis, Oregon, United States of America; Ordway Research Institute, United States of America

## Abstract

Cytokinesis in animal cells requires the assembly of an actomyosin contractile ring to cleave the cell. The ring is highly dynamic; it assembles and disassembles during each cell cleavage, resulting in the recurrent redistribution of actin. To investigate this process in grasshopper spermatocytes, we mechanically manipulated the spindle to induce actin redistribution into ectopic contractile rings, around reassembled lateral spindles. To enhance visualization of actin, we folded the spindle at its equator to convert the remnants of the partially assembled ring into a concentrated source of actin. Filaments from the disintegrating ring aligned along reorganizing spindle microtubules, suggesting that their incorporation into the new ring was mediated by microtubules. We tracked incorporation by speckling actin filaments with Qdots and/or labeling them with Alexa 488-phalloidin. The pattern of movement implied that actin was transported along spindle microtubules, before entering the ring. By double-labeling dividing cells, we imaged actin filaments moving along microtubules near the contractile ring. Together, our findings indicate that in one mechanism of actin redistribution, actin filaments are transported along spindle microtubule tracks in a plus-end–directed fashion. After reaching the spindle midzone, the filaments could be transported laterally to the ring. Notably, actin filaments undergo a dramatic trajectory change as they enter the ring, implying the existence of a pulling force. Two other mechanisms of actin redistribution, cortical flow and de novo assembly, are also present in grasshopper, suggesting that actin converges at the nascent contractile ring from diffuse sources within the cytoplasm and cortex, mediated by spindle microtubules.

## Introduction

Cytokinesis in animal cells occurs upon constriction of the contractile ring, a dynamic structure composed largely of actin and myosin filaments (reviewed in [1]–[5]). This dynamic ring is assembled and disassembled once per cell division – leading to the frequent redistribution of actin within the cell. Recently, contractile ring assembly in fission yeast has generated much interest (e.g., [6]–[9]), and different models have been proposed [10]–[12]. These models may describe parallel pathways for redistribution of actin into the contractile ring [13].

Molecular connections between the ring and spindle apparatus (e.g., [14]–[16]) could provide the basis for understanding microtubule-mediated redistribution of contractile elements into the nascent ring. However, this redistribution has generally been investigated in larger cell types. In silkworm spermatocytes and cultured mammalian cells, dynamic spindle and astral microtubules have been shown to induce the flow of preformed actin filaments from the polar cortex to the equatorial cortex, where the ring is built [17], [18]. Furthermore, in silkworm spermatocytes, actin filaments are also assembled de novo at the plus ends of central spindle microtubules [17]. Aggregates of these actin filaments are transported laterally to the nascent cortical ring by the bundled microtubules. This de novo synthesis continues even as the actin-tipped microtubules splay toward, and ultimately reach, the equatorial cortex [17]. Thus, a contractile ring can be built by recruitment of preformed actin filaments [10], [17]–[19], as well as by de novo assembly [6], [7], [17] – and the mechanisms need not be mutually exclusive [13], [17], [20]. Both the spindle and the contractile ring contain numerous nonstructural proteins [4], [5], [21]; some are involved in signaling between spindle and ring, others may recruit cytokinetic components to their appropriate location and/or retain them there. For instance, the kinesin component of centralspindlin may travel on stabilized spindle microtubules to transport its cargo to the equatorial cortex (as modeled in [22]).

Murthy and Wadsworth demonstrated that actin filaments within the contractile ring are dynamic in an epithelial cell line that stably expressed GFP-actin [23]. By combining FRAP (fluorescence recovery after photobleaching) techniques with pharmacological perturbation, they showed that actin filaments were dynamic both spatially and at the subunit level. That is, movement of fluorescent signal into (and out of) the photobleached ring was accompanied by the turnover of filaments within the ring during recovery of fluorescence. Although the resolution precluded tracking individual actin filaments, bulk flow of actin could be monitored using changes in fluorescence per unit area over time.

Clearly assembly of the contractile ring is critical for cytokinesis, but disassembly of the constricting ring is also important. Myosin II plays a role in both processes (e.g., [23]–[25]). Using cryo-transmission electron microscopy, Haviv et al. observed the in vitro disassembly of actin bundles by myosin II motor proteins in a concentration dependent manner. The authors proposed that disassembly of the contractile ring could be regulated by “fine tuning the local concentration/activity of myosin II motors” [25].

In this paper, we characterized mechanisms of actin redistribution in spermatocytes of the grasshopper *Melanopus femurrubrum*. Using the spindle as a tool to manipulate actin redistribution, we engineered microtubule-mediated movement of actin filaments by mechanically remodeling the spindle, which was readily maneuverable by microneedle. These experiments led us to hypothesize that actin filaments can travel along spindle microtubules to the spindle equator. To test this hypothesis, we labeled actin filaments with phalloidin conjugated Quantum dots (Qdots) and/or Alexa 488 Phalloidin, and tracked their movement in and out of the nascent contractile ring. The pattern of filament movement further implied that actin was transported along spindle microtubules, before being drawn into the ring. Indeed, in double-labeled dividing cells, actin filaments could be seen moving along microtubules toward the contractile ring. Two other mechanisms of actin redistribution, cortical flow and de novo assembly, were also shown to be present in grasshopper. Together, our results suggested that actin converges at the nascent contractile ring from diffuse sources within the cytoplasm and cortex, mediated by spindle microtubules.

## Results

### Mechanically induced reorganization of spindle microtubules mediated actin redistribution

**Figure 1:**
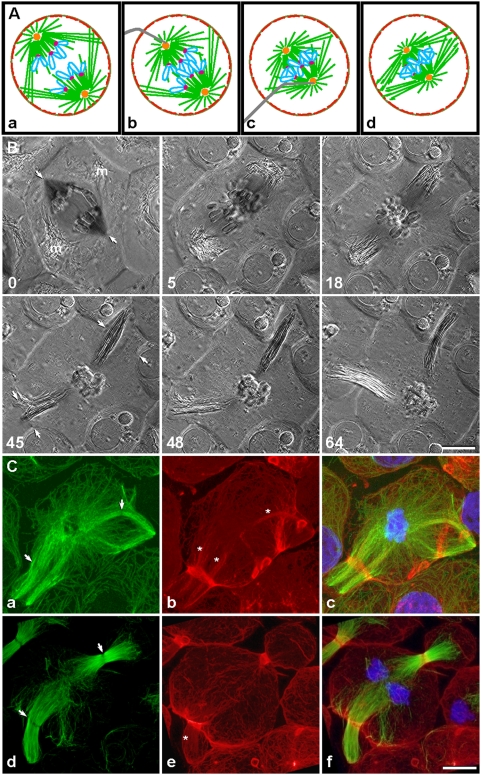
Mechanically induced reorganization of microtubules mediated actin redistribution into ectopic contractile rings. (A, a–d) The diagram shows how an anaphase spindle can be mechanically collapsed by pushing the spindle poles together, using a micromanipulation needle. The procedure results in ectopic cleavage in two locations, as shown in (B). Microtubules, green; chromosomes, blue; kinetochores, magenta; centrosomes, orange; cortical actin, red; needle, gray. (B) Polarization microscopy sequence of ectopic cleavage, with time shown in minutes. Following anaphase onset (0 min, arrows show direction of impending collapse; m, spindle-associated mitochondria; also see Video S1), the spindle was collapsed with a microneedle, resulting in lateral growth of microtubules that dislodged the mitochondria (5 min). On opposite sides of the cell, these microtubules reorganized into two lateral spindles, while the displaced mitochondria appeared to rebundle with the microtubules (18 min). Cleavage furrows initiated simultaneously around both of the continuously reorganizing lateral spindles (45 min, arrows). Ultimately, each furrow ingressed around the approximate midpoint of its spindle (48 min), producing two anucleate membrane pockets (64 min). (C) Distribution of microtubules (green), actin filaments (red), and chromosomes (blue) in cells fixed at furrow initiation (a–c) and ingression (d–f) showed that each lateral spindle was in fact an independent bipolar spindle complete with a midzone (a and d, arrows). Bands of actin filaments (b and e), presumably contractile rings, encircled the midzone regions, while another subset of actin filaments (marked by asterisks) appeared to colocalize with spindle microtubules. Remnants of contractile rings from previous cell cleavages, i.e., “cell division scars”, were also present (b and e; small circles or ovals at cortex). Bars, 10 µm.

In 1949, Hans Ris showed that a pair of ectopic furrows could be induced around reorganized lateral spindles in grasshopper spermatocytes, if chromosomes became too “sticky” to segregate due to hypertonic medium, X-rays, or elevated temperatures [26]. This intriguing phenomenon prompted us to test our proposition that the reorganizing microtubules could mediate redistribution of actin into ectopic contractile rings. Instead of relying on inseparable chromosomes in unhealthy cells, we used a microneedle to mechanically collapse an anaphase spindle, thereby bringing the homologous chromosomes permanently back together (Fig. 1A, a–d). When chromosomes were thus prevented from separating during anaphase in primary cultures of flattened spermatocytes, equatorial furrow formation was disrupted by spontaneous reorganization of microtubules into two lateral spindles originating from the collapsed spindle (Fig. 1A,d). Immediately prior to collapse of the spindle (Fig. 1B, 0 min, arrows show direction of impending collapse), mitochondria were seen flanking the spindle midzone region. The reorganization of the collapsed spindle was captured in a polarization microscopy sequence (Fig. 1B; Video S1; *n* = 12), which showed the mitochondria being pushed outward by two bundles of laterally protruding microtubules (Fig. 1B; 5 min; microtubules appeared as black fibers). Shortly after spindle-collapse, the mitochondria, bundled with the microtubules, extended toward the cell periphery, while the chromosomes and asters remained clustered in the interior (Fig. 1B; 18 min). The lengthening of the microtubule bundles (i.e., the newly formed lateral spindles) was accommodated by a concurrent bud-like distortion and expansion of the cell membrane. Ectopic cleavage furrows initiated simultaneously near the distal end of each lateral spindle (Fig. 1B; 45 min, arrows), and the two furrows ingressed synchronously as each spindle extended farther into the nascent membrane pocket (Fig. 1B; 48 min). By about an hour after spindle collapse, the ingressing furrows had constricted almost to completion around the approximate midpoint of each lateral spindle (Fig. 1B; 64 min), producing two anucleate membrane pockets partially attached to the mother cell [17], [27].

Cells fixed and stained at furrow initiation (Fig. 1C, a–c; *n* = 5) or during ingression (Fig. 1C, d–f; *n* = 5) contained lateral spindles comprised largely of bundled bipolar microtubule arrays. The lack of staining of a narrow band within each spindle suggested the presence of a midzone that excluded anti-tubulin antibodies (Fig. 1C; a and d, arrows) [27]. Actin filaments (Fig. 1C, b–c and e–f) accumulated in a band that precisely encircled the midzone (Fig. 1C, a and d, arrows), forming a well-organized contractile ring. In addition, some actin filaments colocalized with spindle microtubules, particularly at the furrow initiation stage (marked by asterisks in Fig. 1C, b), but less prominently at later stages (marked by an asterisk in Fig. 1C, e). Within the spindle, actin filaments appeared to align parallel to the microtubule bundles. This colocalization of spindle microtubules and actin filaments suggested a physical association between the two, raising the possibility that the actin filaments could travel along microtubule tracks toward the incipient contractile ring. The apparent decrease in costaining seen in later stages was consistent with the idea that the actin may have reached the contractile ring.

### Spindle folding generated a concentrated source of actin for redistribution

**Figure 2:**
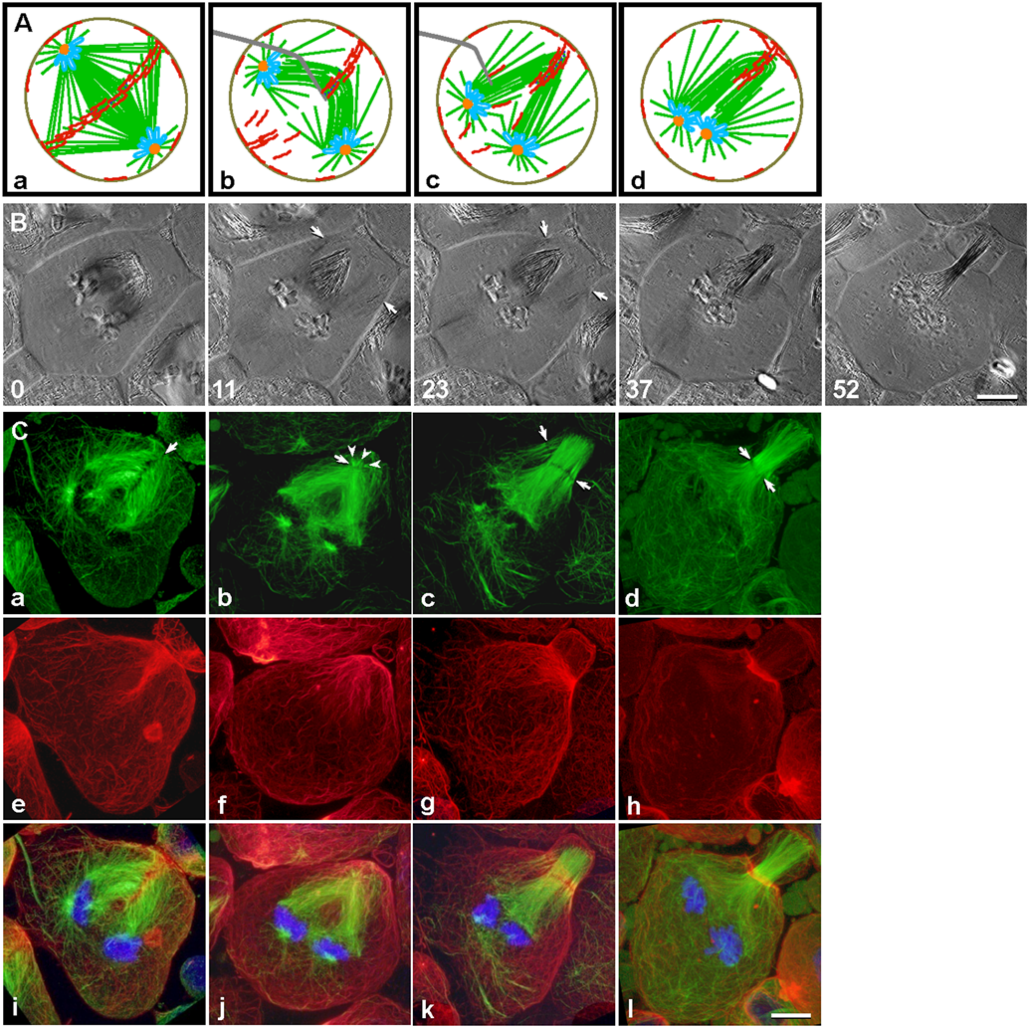
Spindle folding generated a concentrated source of actin for building a new contractile ring. (A) The central spindle can be folded using a microneedle, before furrow formation. Color scheme, as in Fig. 1A. (B) Polarization microscopy sequence (Video S2). After folding (0 min), the mechanically generated monopolar spindle reorganized into a bipolar spindle. Microtubules from the pair of spindle poles radiated toward the cell cortex (11 min, arrows) where a furrow initiated (23 min, arrows). The furrow ultimately ingressed at the equator of the spindle (37–52 min), after realignment of the spindle midzone. (C) Distribution of microtubules (green), actin filaments (red), and chromosomes (blue) in cells fixed at stages similar to those in (B). Upon folding of the spindle by micromanipulation, the deformed midzone (a, arrow) and remnants of the contractile ring (e and i) remained relatively organized. As central spindle microtubules reorganized, the original midzone disappeared (b). Concurrently, actin filaments dispersed from the disintegrating ring, oriented in apparent alignment with central spindle microtubules (f and j). Meanwhile, a few very short microtubule bundles (b, arrowheads) emerged at the spindle tip and initiated formation of a new midzone, transverse to the new spindle axis (b, arrow). Around the time of furrow initiation, reorganization of the elongated bundles created a new “pole” (c), thereby establishing a bipolar spindle with a broad midzone (c, arrows). Actin filaments appeared to disassociate from spindle microtubules as they entered the new contractile ring (g and k). The furrow ingressed as microtubules at the distal pole elongated (c–d, green), shifting the midzone (c–d, arrows) and the contractile ring (g–h and k–l) toward the spindle equator. Bars, 10 µm.

In a variation on our spindle-collapsing experiment (Fig. 1), we folded a telophase spindle to induce actin redistribution into a new ring, around a reassembled spindle (Fig. 2). Although both operations brought the two spindle poles into proximity, collapsing did so by pushing segregating anaphase chromosomes back together, whereas folding bent the spindle at its equator to swing the segregated telophase chromosomes toward each other (compare Figs. 1A and 2A). Note that part of the assembling contractile ring was destroyed as the spindle equator was pushed away from the cortex by a microneedle (Fig. 2A, b). We reasoned that folding the spindle would enhance visualization of actin redistribution, because the remnants of the original ring would serve as a more concentrated source of actin, available for building the new ring. By scaling up redistribution of actin, we hoped to gain insight as to how microtubules move actin into the contractile ring.

As seen in a polarization microscopy sequence (Fig. 2B; Video S2, *n* = 15), the microtubules of the newly folded central spindle (0 min, equivalent to schematic 2A, d) began to reorganize, lengthening from the joint poles toward the cortex (0–11 min, arrows). Furrow initiation coincided approximately with the arrival of spindle microtubules at the cortex (23 min, arrows). The nascent furrow encircled the long axis of the microtubule bundle, near its distal end. The plane of this furrow was perpendicular to the one that would have formed in an undisturbed cell. As the distal end of the elongating spindle extended towards the cell membrane, the midzone of the new spindle was concomitantly repositioned to the midpoint of the spindle (23 min onward). Furrow ingression and formation of the anucleate membrane pocket appeared similar in the manipulated cells, regardless of whether the spindle was folded (Fig. 2) or collapsed (Fig. 1).

To track the relative redistribution of actin filaments and microtubules during spindle reorganization, cells were fixed at stages similar to those in the Figure 2B sequence (Fig. 2C; *n* = 5 for each stage shown). After the spindle was folded, the displaced midzone appeared well organized initially, but soon disappeared as spindle microtubules reorganized (Fig. 2C, a, arrow). Although the contractile ring had been broken by micromanipulation, many actin filaments remained in the region of the midzone before the ring disintegrated (Fig. 2C, a, e and i). Concurrently with the reorganization of spindle microtubules (Fig. 2C, b), pre-existing actin filaments dispersed from the disintegrating contractile ring and appeared to follow the distribution of microtubules (Fig. 2C, b, f and j). This apparent colocalization of actin filaments and microtubules again suggested a physical association between the two, as had been seen in Fig. 1C. Although it was possible that the actin filaments could be bound to a spindle matrix [28], the orientation of the filaments was not random. Their coalignment with the microtubules lent credence to a physical association. Furthermore, the intensity of fluorescence in the new contractile ring increased as the remnants of the original contractile ring disappeared, consistent with the idea that the actin filaments moved in the direction of the contractile ring. Notably, several very short microtubule bundles (Fig. 2C, b, arrowheads) assembled at the distal end of the nascent spindle and established a new midzone-like structure that transected the folded bundles of microtubules (Fig. 2C, b, arrow). At furrow initiation, numerous elongated microtubule bundles organized into an anastral pole at the cell periphery (Fig. 2C, c). Thus, the manipulated cells were capable of establishing what appeared to be a new bipolar spindle, complete with a midzone (Fig. 2C, c, arrows).

Actin filaments, initially aligned along microtubules, ultimately reoriented by about 90° to merge into the incipient contractile ring, which snugly encircled the broad midzone's periphery (Fig. 2C, g and k, red). During furrow ingression, the spindle continued to lengthen in the direction of the distal pole (Fig. 2C, c–d, green), shifting the midzone and its associated contractile ring (Fig. 2C, g–h and k–l, red) progressively closer to the spindle midpoint (Fig. 2C, c–d, arrows). Measurements made in living cells (*n* = 7) showed that microtubules at the distal pole grew from 2.3±0.7 µm at furrow initiation to 12.1±1.8 µm following ingression, approximately matching the length of microtubules at the original pole. This observation was similar to our previous study of reorganization and elongation in cells containing cut half-spindles [29]. A spindle induced by folding is similar to a lateral spindle: both structures contain a newly assembled half spindle at the distal end, whereas their chromosomes, original spindle poles, and astral microtubules are located in the central region of the cell. Thus, conclusions deduced from spindle-folding experiments would be expected to apply as well to spindle reorganization and actin redistribution of the sort observed in spindle-collapsing experiments (as in Fig. 1).

### Tracking actin redistribution during contractile ring assembly

**Figure 3:**
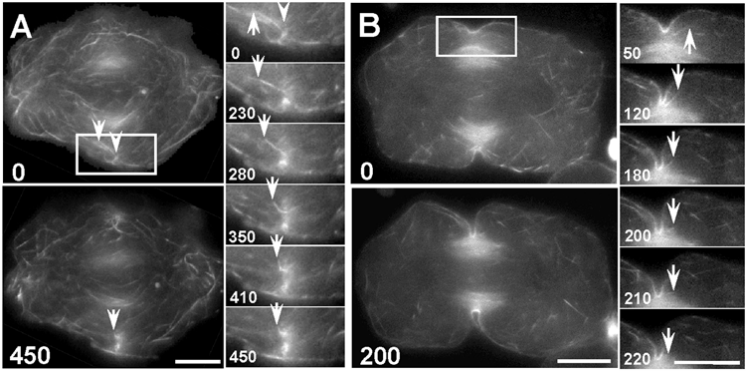
Incorporation of actin filaments into the contractile ring during furrow induction and ingression. (A) Surface view of induction. Actin filaments were labeled by microinjection with Alexa 488-phalloidin and imaged every 10 seconds. The large panels show overall actin redistribution over time. The small panels show the dynamics of a single actin filament (arrows), as seen in selected time-lapse images within the boxed region of interest (Video S3). Immediately prior to furrow initiation (0 sec image), some cortical actin filaments began to move toward the spindle equator where they coalesced into nascent bundles of the emerging contractile ring (arrowhead). The arrows mark the end of a filament of interest, discernible at this focal plane. The filament moved toward the equator, underwent a sharp change in trajectory, and disappeared into the emerging contractile ring. The unlabeled spindle was flanked by microtubule-associated, autofluorescent mitochondria (also present in (B)) that hampered visualization of actin filaments in the vicinity. Time in seconds. Bars, 10 µm. (B) Midplane view of ingression. Actin filaments were labeled and observed as described in (A) except imaged at the midplane of the cell to follow furrow ingression. In the large panels, both labeled actin filaments and autofluorescent mitochondria could be seen. During furrow ingression (arrowheads), actin filaments continued to move toward and incorporate into the contractile ring, presumably along spindle microtubules. The small panels (selected from Video S4; within the boxed region) show the dynamics of a single actin filament (arrows) moving into the ingressing contractile ring. Time in seconds. Bars, 10 µm.

**Figure 4:**
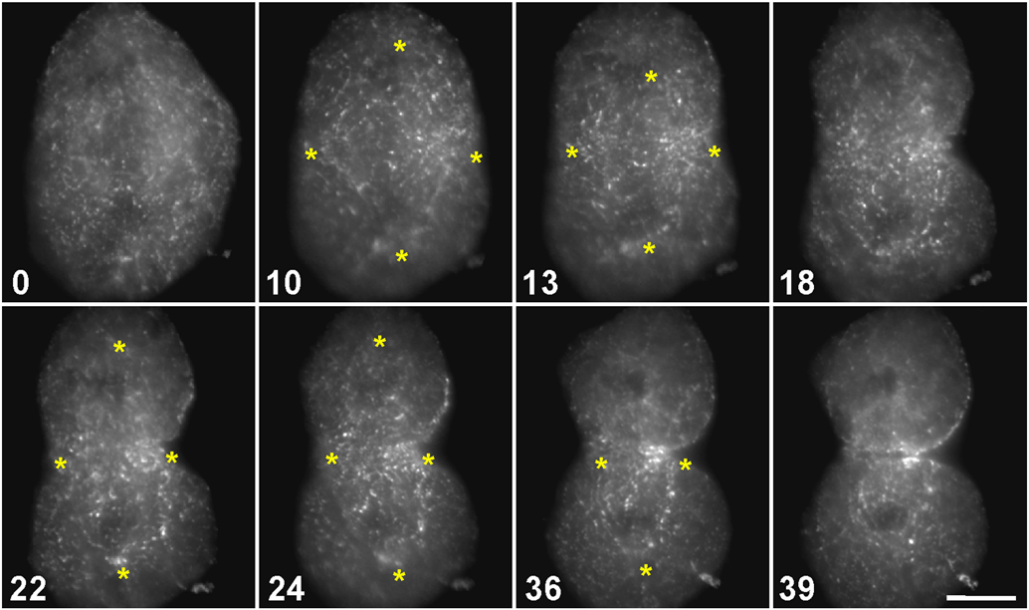
Redistribution of actin filaments labeled by microinjection of Qdot 655-Phalloidin. At the onset of cytokinesis (10), Qdot-decorated actin filaments formed a pattern vaguely mirroring that of the spindle (10–13), reminiscent of the distribution of central spindle microtubules (as in Video S5). The Qdots gradually cleared from the non-equatorial regions and accumulated at the cleavage furrow (18 onward) as cytokinesis proceeded. Time in minutes. Bar, 10 µm.

**Figure 5 pone-0004892-g005:**
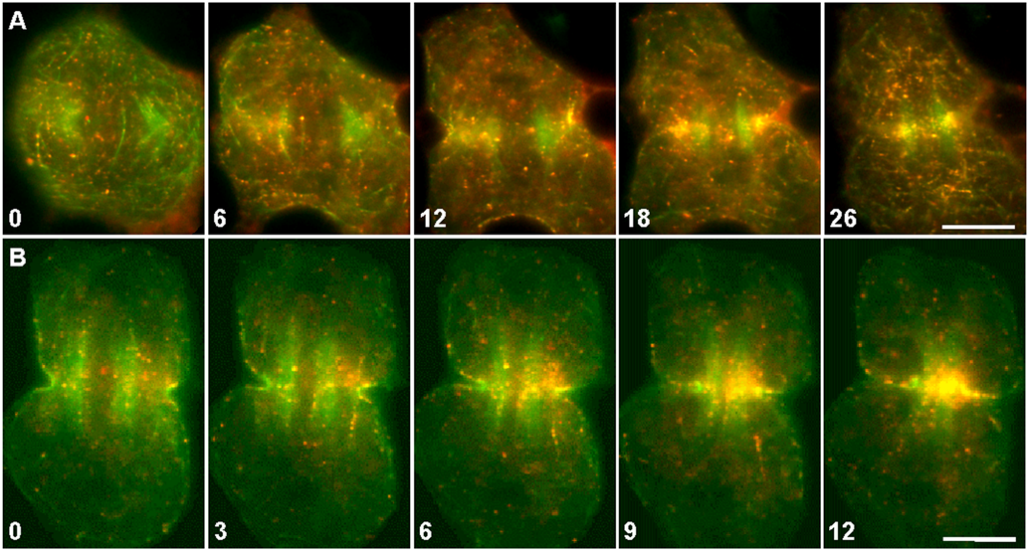
Redistribution of actin filaments labeled by Alexa 488-Phalloidin and Qdot 655-Phalloidin. (A–B) Actin filaments (green) speckled with Qdots (red) continuously merged into the contractile ring during furrow induction (A; Video S6) and furrow ingression (B; Video S7). Occasionally, Qdot-decorated actin filaments could be seen moving away from the contractile ring (B, 3–9). The unlabeled spindles were flanked by a pair of green bundles that contained microtubule-associated, autofluorescent mitochondria. Time in minutes. Bar, 10 µm.

**Figure 6:**
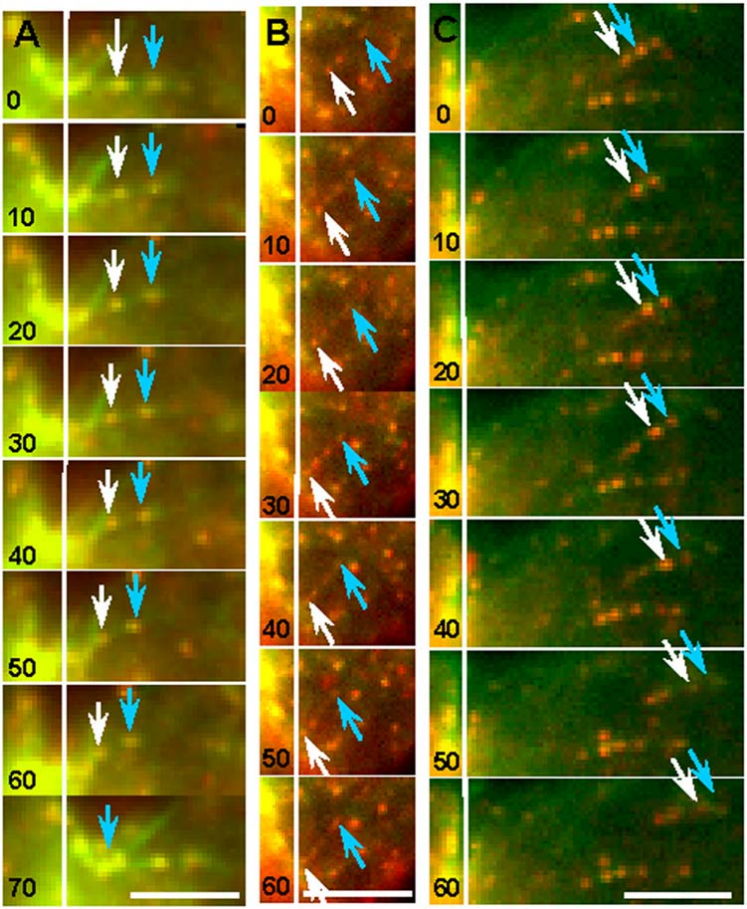
Tracking individual actin filaments labeled by Alexa 488-Phalloidin and speckled with Qdot 655-Phalloidin. As shown in the kymographs (Videos S8, S9, S10), actin filaments could be seen moving into the cleavage furrow (A, B) and moving away from the furrow (C). Arrows depict Qdot-marked reference points on the actin filaments, and a white vertical line marks the location of each cleavage furrow. Time in seconds. Bar, 10 µm.

The contractile ring assembles during cytokinesis and remains highly dynamic as it constricts the cell (e.g., [12], [20], [23]). Although preexisting and newly polymerized actin filaments or bundles, as well as actin subunits, may all contribute to the ring dynamics, it is unclear whether preformed actin filaments are static or dynamic as they enter into the nascent contractile ring. Furthermore, it is unclear by what mechanism the filaments enter the ring. Are they released from the microtubules in the vicinity of the ring, and do they then passively drift toward the ring? Or are they actively pulled in by a component of the contractile ring? Finally, we wanted to determine whether the behavior of actin in live cells was consistent with our hypothesis that actin could travel along spindle microtubules toward the contractile ring. To address these questions, we labeled the actin filaments in dividing cells in a variety of ways (Figs. 3, 4, 5, 6).

First, we labeled the actin filaments by microinjecting cells with Alexa 488-phalloidin. The actin filaments were followed using time-lapse microscopy, focusing on the cortical region during furrow induction (Fig. 3A; Video S3; *n* = 26 cells) and the midplane during furrow ingression (Fig. 3B; Video S4; *n* = 28 cells). The filament highlighted in Fig. 3A approached the ring with its long axis approximately perpendicular to the plane of the ring, an orientation consistent with its hypothesized movement along spindle microtubules. Notably, upon entering the ring, the filament changed its trajectory abruptly, as if it were pulled into the ring. Incorporation of actin filaments into the contractile ring was also observed during furrow ingression (Fig. 3B). The trajectory change of the incorporating filament was not visible at the midplane as the filament would be expected to turn perpendicularly with respect to the plane of view while entering the ring.

Next, the actin filaments were labeled by microinjection with trace amounts of Qdot 655 phalloidin conjugates (Fig. 4; Video S5; *n* = 3). Qdots are nanocrystals that function as highly photostable fluorophores. When present at a very low concentration, Qdot 655 phalloidin bound stochastically to actin filaments, generating bright, photostable speckles along the actin filaments. Over time, the Qdots became concentrated in the vicinity of the contractile ring. During their movement towards the ring, a very subtle pattern could be seen: the dots were slightly more prevalent in the region of the central spindle, i.e., in a large diamond-shaped region between the two spindle poles. (The poles were marked approximately by dark circles, due to signal exclusion from the region occupied by the segregated chromosomes, and the vertices were depicted by asterisks.) This diamond was most obvious in the panel for the 10-minute time point in Fig. 4, and its similarity in shape to the bipolar spindle was consistent with the idea that Qdot-labeled actin filaments were binding to the spindle. Also, the filaments may be skewed diagonally, parallel to the edges of the diamond, as if aligning along the microtubules. We surmised that this pattern was not distinct because only certain labeled filaments would be spindle-associated. Others filaments would be cortical, with the relative proportion of each depending on the location and depth of the selected focal plane. Nonetheless, the speckled actin filaments were progressively cleared from the cytoplasm and accumulated at the spindle equator, suggesting the presence of a directional bulk flow of cytoplasmic actin (Fig. 4; also Figs. 5A–B and Videos S5, S6, S7). As cytokinesis proceeded, these filaments appeared to merge into a belt of actin bundles within the ring. Based on the spindle-shaped localization pattern of a subset of the Qdots, along with their directionally biased movement, we speculated that some actin filaments could be traveling down the microtubules of the central spindle toward the contractile ring.

Actin filaments were also colabeled by microinjection with trace amounts of Alexa 488 phalloidin and Qdot 655 phalloidin conjugates, which generated red, photo-stable speckles along the green Alexa 488 phalloidin-stained actin filaments (Figs. 5A–B, 6A–C; Videos S6, S7, S8, S9, S10, respectively; *n* = 4 cells, each showing numerous actin filaments moving towards, and occasionally away from, the furrow). These quantum dots provide precise reference points for determining the change in length of individual actin filaments, and for monitoring their movement relative to other filaments or cellular constituents during cytokinesis. We observed that intact actin filaments could enter the ring during assembly (Fig. 6 A–B) and exit from the ring during disassembly (Fig. 6C). Thus, the length of at least some of these filaments appeared stable in the vicinity of the ring – confirming that the ring was not built exclusively by on-site, de novo-assembly, nor was it disassembled exclusively by depolymerization within the ring. In addition, the filaments did not appear to be undergoing significant levels of treadmilling.

### Movement of actin filaments along spindle microtubules toward the cortex

**Figure 7:**
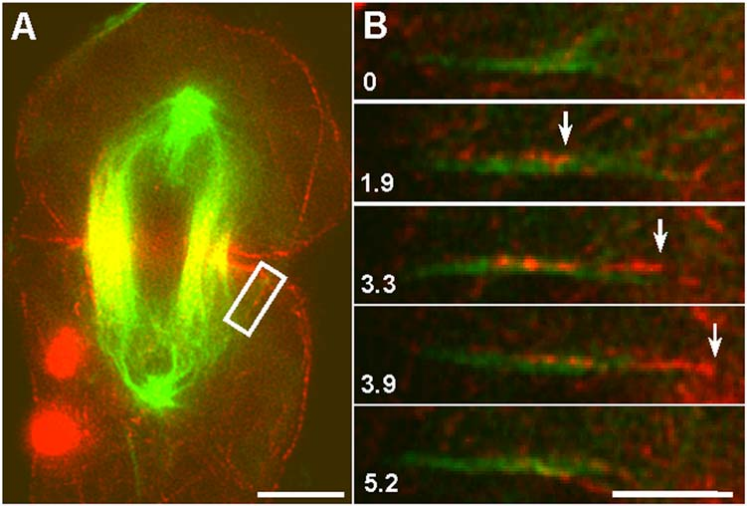
The movement of an actin filament along sparsely distributed spindle microtubules during furrow ingression. For fluorescent speckle microscopy, microtubules (false-colored green) and actin filaments (false-colored red) were labeled by microinjection with Rhodamine-tubulin and a minute amount (∼0.2 µM) of Alexa 488-phalloidin, respectively. (A) A selected image from a time lapse series showing a dividing cell in which a single, clearly visible microtubule (boxed) happened to be isolated from the rest of the central spindle. Bar, 10 µm. (B) Sequential images of the boxed region, containing the cell cortex and the microtubule of interest, were enlarged and reoriented, with the microtubule horizontal and the cell cortex toward the right. A speckled actin filament (or bundle) appeared to move along a single microtubule toward the cortex. Bar, 5 µm.

Ideally, we would have liked to test by direct observation our hypothesis that actin filaments can use microtubules in the central spindle as tracks on which to travel toward the contractile ring. However, the high density of microtubules in the central spindle and the presence of mitochondria were major impediments. Instead, we focused our attention on stray spindle microtubules that grew toward the ingressing furrow in less densely populated regions. The microtubules (false-colored green) were labeled by microinjection with Rhodamine-tubulin in anaphase. To create reference points for following individual filaments, actin filaments (false-colored red) were speckled by microinjection with a minute amount of Alexa 488-phalloidin (∼0.2 µM estimated intracellular concentration). An analysis of time-lapse sequences recorded in 18 cells showed that in each case, a speckled actin filament moved along the microtubule towards the cortex (Fig. 7A–B; *n* = 18 cells, with at least one observation per cell). Our observations support the hypothesis that actin filaments can use microtubule tracks as a means to approach the contractile ring. Because only actin was speckled, it was unclear whether the microtubules remained static or dynamic during the course of the observations. Although the origin of these microtubules remains to be defined, they could nonetheless function as a bridge between spindle pole and cortex, along which actin filaments could move. If actin filaments were transported to the cortex outside of the equatorial region, cortical flow could deliver them to the ring [17], [30].

### Cortical flow and de novo assembly of actin filaments in grasshopper spermatocytes

**Figure 8:**
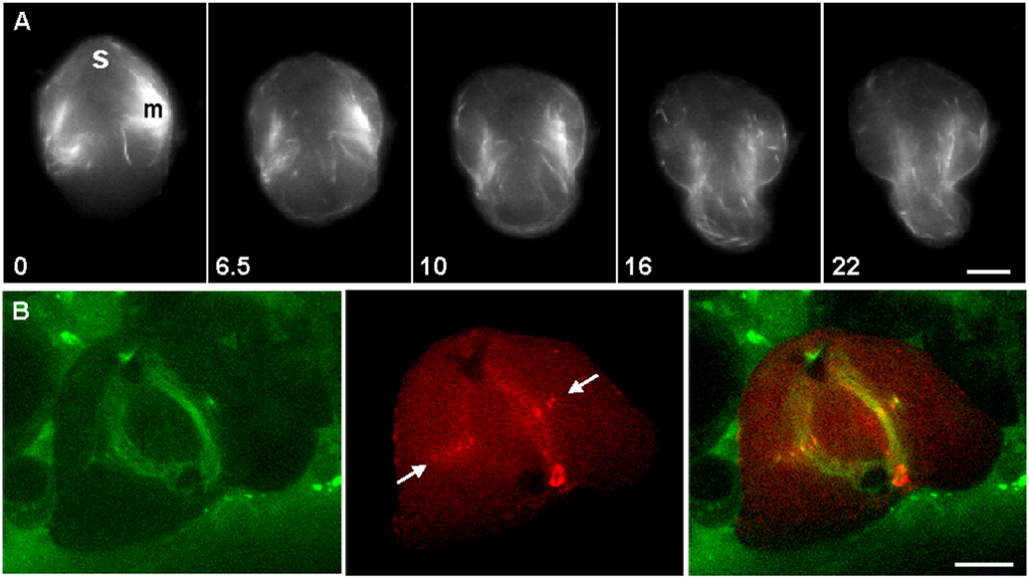
Cortical flow and de novo synthesis of actin filaments in grasshopper spermatocytes. (A) Time-lapse sequence of actin filaments undergoing cortical flow (from Video S11). Flow of actin was induced by collapsing the spindle and repositioning it near one side of the cortex. The final location of the spindle is marked by “sp”. Actin filaments were labeled by microinjection with Alexa 488 phalloidin. Note the clearing over time of the region closest to the spindle, as the actin flows to the cortex on the opposite side of the cell. Spindle-associated mitochondria were seen as a pair of large bright patches (labeled “m”) that autofluoresced in the FITC channel. Because mitochondria were localized to the cytoplasm, their displacement was not related to cortical flow, but rather was driven by the elongation of dynamic microtubules with which they were associated. Time in minutes. (B) A confocal micrograph showing actin aggregates (red, marked by arrows) localized to the tips of bundled microtubules (green) at the spindle midzone. These aggregates were strikingly similar in their location, timing of appearance and morphology to aggregates found in silkworm spermatocytes [17], which have been shown to be assembled de novo at the midzone. Actin was labeled by microinjection of rhodamine phalloidin during anaphase, and microtubules were labeled with paclitaxel green. A cell division scar (red circle, lower edge of cell) was visible. Bars, 10 µm.

In addition to transport of actin along microtubules as one mechanism for redistributing actin toward the contractile ring, actin redistribution in grasshopper spermatocytes could occur by cortical flow as it does in silkworm spermatocytes [17] and cultured mammalian cells [18], [31]. A comparison of panels e–g (Fig. 2C) with panel h suggests the existence of cortical flow, as a significant clearing of actin (presumably cortical) occurred from the region outside of the cortical ring. To conclusively demonstrate the existence of cortical flow in grasshopper spermatocytes, we repeated an experiment previously performed in silkworm spermatocytes [17]. By collapsing the spindle with a microneedle and repositioning it near the cortex, we induced cortical flow of Alexa 488 phalloidin-labeled actin away from the spindle (Fig. 8A; Video S11; *n* = 10). The movement of actin filaments, either individual or bundled, was readily apparent in a representative cell (panel 8A). The pair of large bright patches (labeled “m”) was generated by two bundles of spindle-associated mitochondria, which autofluoresced in the FITC channel and shifted when microtubules in the reorganizing spindle elongated to drive cortical flow. Thus, even though the mitochondria also moved away from the spindle, their motion was associated only indirectly with cortical flow.

In addition to documenting cortical flow in silkworm spermatocytes, we had previously demonstrated redistribution of actin into the contractile ring by means of a two-part mechanism [17]. In this mechanism, actin aggregates are assembled de novo at the plus ends of microtubules at the midzone of the central spindle. The aggregates are then transported from the midzone to the nascent contractile ring, riding on the tips of microtubule bundles as the spindle halves splay out laterally. We wanted to determine whether de novo assembly could also be present in grasshopper spermatocytes. Flattened grasshopper spermatocytes in anaphase were microinjected with rhodamine phalloidin to label the polymerized actin and stained with fluorophore-conjugated paclitaxel (Tubulin Tracker) to label and stabilize spindle microtubules. Phalloidin-labeled actin was localized to the plus ends of bundled microtubules in the central spindle (Fig. 8D; *n* = 3), forming punctate dots that closely resembled the actin aggregates seen in silkworm spermatocytes [17]. The location, timing and appearance of the aggregates in grasshopper spermatocytes suggested that de novo assembly might be a common mechanism used for actin redistribution during contractile ring assembly in insect spermatocytes. Furthermore, recently published data ([32]; Fig. 3E) showing actin aggregates at the spindle midzone could be interpreted as evidence to suggest the existence of this mechanism in mouse oocytes.

### Cell division does not require the presence of spindle-associated mitochondria

**Figure 9:**
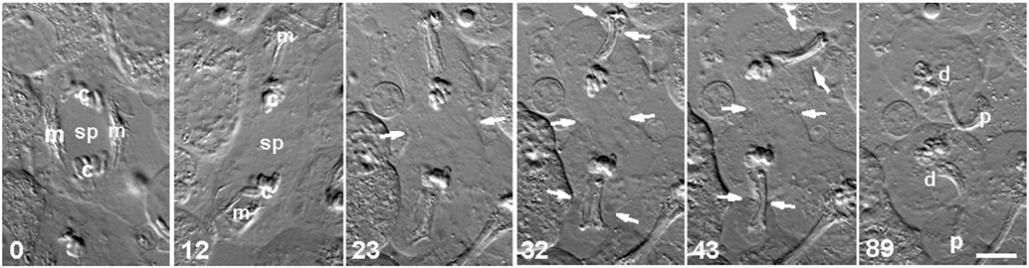
Cell division did not require the presence of spindle-associated mitochondria. A time-lapse sequence of DIC images. During anaphase (0 min), both groups of spindle-associated mitochondria (m) that flanked the central spindle (sp) were mechanically repositioned distal to opposing sets of segregating chromosomes (c; 12 min). Although the equatorial region was thus cleared of mitochondria, cleavage at the spindle equator was not inhibited (12 min onward). Arrows show the locations of the cleavage furrows: at the equator, and in two ectopic locations induced by the relocated, mitochondria-associated bundles of microtubules. The furrows produced two daughter cells (d), each associated with an anucleate membrane pocket (p). Time in minutes. Bar, 10 µm.

Recent research on mitochondrial function and morphology has shown that mitochondria are more than just the cell's ‘powerhouse’ (reviewed in [33]). For example, the mitochondrial membrane provides a surface on which Arp2/3 can nucleate formation of actin cables in yeast. Thus, mitochondria can promote their own anterograde transport along actin cables, apparently by recruiting their own tracks. Furthermore, the mitochondria (“m” in Fig. 9) in grasshopper spermatocytes are highly elongated and flank the central spindle. This spermatocyte-specific mitochondrial distribution, combined with the potential for interaction between actin and mitochondria [34] raised concerns that our observations and interpretations of actin redistribution could be confounded. Specifically, in late anaphase, these spindle-associated mitochondria splay towards the equatorial cortex, thus potentially co-transporting any associated actin filaments to the incipient contractile ring. Therefore, we hoped to determine whether the spindle-associated mitochondria played a specialized role in cytokinesis, in addition to their more general role in energy production. For example, spindle-associated mitochondria could conceivably play a role in actin assembly and/or delivery to the contractile ring. Alternatively, as mitochondria are known to function in multiple signal transduction cascades, perhaps spindle-associated mitochondria helped to signal the location or timing of furrowing.

To determine whether these mitochondria were critical for induction or ingression of furrowing, we wanted to find out if removing them was a viable option. We used a microneedle to maneuver the two bundles of mitochondria-associated microtubules away from the central spindle during anaphase; they were repositioned distal to the segregating chromosomes (Fig. 9, 0–12 min; *n* = 5). More specifically, one of the pair of mitochondria-associated bundles was severed at one spindle pole, and pivoted by 180° around the opposite spindle pole, thus creating a narrow, spindle-length bipolar structure attached at one end to one of the original spindle poles. This operation was repeated with the remaining mitochondria-associated bundle, this time severing near the other pole. The inner portion of the central spindle was left intact. In addition, the entire spindle was rotated, thus preventing furrow induction based on preexisting furrow cues. Three furrows were induced: one at the equatorial cortex (now free of mitochondria), and one around each of the relocated, bundled microtubules, cleaving the cell into two daughter cells and two anucleate membrane pockets (Fig. 9, 23 min onward, arrows). This simple experiment showed that colocalization of mitochondria and spindle microtubules (at least from anaphase onward) was not required for any readily detectable aspect of furrow induction or ingression. Although the mitochondria-associated microtubule bundles could induce furrows as well [27], the presence of mitochondria does not confound our interpretation of the data on the role of spindle microtubules.

## Discussion

We hypothesized that one way in which cytoplasmic actin filaments can be redistributed during cytokinesis is by transport along the spindle microtubules. Using mechanical micromanipulation to remodel the spindle, we showed that spindle microtubules could reassemble into lateral or asymmetric spindles, depending on whether the spindle was remodeled by collapsing or by folding. These self-assembled spindles were composed of what appeared to be bipolar microtubule arrays, with a midzone-like region resistant to staining with an anti-tubulin antibody. The micromanipulation experiments also allowed us to generate a concentrated source of actin (i.e., the remnants of the original contractile ring) and to observe, in sequentially fixed cells, its redistribution into a new ring. The actin filaments colocalized with the microtubules in the reassembling spindles and appeared to travel toward the plus ends. By tracking actin filaments that were speckled with Qdots and/or labeled with Alexa 488-phalloidin in dividing cells, we showed that the pattern of actin movement was consistent with actin redistribution from the micromanipulated cells, and that the actin filaments appeared to be pulled into the ring. Finally, the strongest support for our hypothesis comes from double-label tracking experiments in which we showed that the filaments not only colocalized with, but also traveled along, spindle microtubules toward the microtubule plus ends to the equatorial cortex.

We believe that all the basic mechanisms of actin redistribution seen in manipulated cells are also present in unperturbed cells. Given our experimental time frame, it seems unlikely that mechanical manipulation can significantly change the biochemical composition of the cell, although the dynamics of actin and microtubule polymerization could certainly be affected. Also, lateral or asymmetric spindles were able to reassemble, in spite of the manipulations. Our manipulations served merely to amplify and highlight the functional interdependence of actin and microtubules during cell division. For example, folding the spindle to artificially concentrate actin, in a region of high microtubule density, served to augment redistribution of actin along microtubule tracks, facilitating visualization.

We have demonstrated that mechanically folded or collapsed spindles can reassemble into what appears to be one or two bipolar spindles, respectively, each with a functional midzone. How might this reassembly take place? Soon after the spindle is folded, several short bundles of microtubules become visible at the distal end of the folded bundle (Fig. 2C, b). The polarity of these microtubules is presumably opposite to that of the microtubules in the main bundle. We propose that the short bundles nucleate a new half spindle [29]. The interior end of the spindle appears stationary over time, and the final length of the reassembled spindle is close to that of the original spindle. Furthermore, the ultimate position of the midzone-like structure closely matches that of the distal end of the newly folded spindle. These observations are consistent with a model in which there is unidirectional elongation and sliding of only the nascent, distal spindle half. The outward sliding of the elongating spindle half would result in the shifting of the midzone toward the spindle midpoint, thus generating symmetrical spindle-halves. We hypothesize that the midzone re-forms, driven by recruitment of midzone proteins, including those recently dissociated from the original midzone, to the region of microtubule overlap.

By tracking both actin and microtubules (Fig. 7B), we provide evidence that actin filaments colocalize with, and move directionally along, microtubules, toward the equator. However, by necessity, that experiment can address only local, small-scale redistribution of actin. Large-scale redistribution of actin is addressed by our Qdot experiments in which speckled actin filaments are progressively cleared from the non-equatorial region and accumulate at the spindle equator, i.e., a directional bulk flow of cytoplasmic actin (Figs. 4–6 and associated videos). This directionally biased flow is consistent with movement of actin along spindle microtubules toward the equator. However, because the Qdots could also label cortical actin filaments, not just the spindle associated ones, some of the observed filaments could be delivered to the equatorial region by the also directionally biased cortical flow. Furthermore, as actin gets assembled de novo in the spindle midzone, it might pick up some previously unbound Qdots, thus appearing to materialize out of nowhere at the equator. These newly labeled filaments would enrich the equatorial signal over time, without actually having moved from pole to equator. Nonetheless, a sizable number of Qdot-labeled filaments, that were undeniably within the spindle proper (e.g., as in Fig. 6A–B), also moved toward the plus ends of spindle microtubules. It is the directional flow of these filaments, the ones populating the interior of the cell, that lends credence to our hypothesis that actin filaments travel down spindle microtubules to the equator. This transport occurs along spindle microtubules in a plus-end–directed fashion, possibly driven by kinesin/myosin V-type hetero-motors as modeled in [35], [36] or by a myosin 10-type motor that binds both actin and microtubules [37]. Upon traveling to the plus ends of the microtubules at the spindle equator, the filaments might complete their journey to the contractile ring using a microtubule-mediated lateral transport mechanism, as do the de novo assembled filaments [17].

We observed sharp changes in trajectory of individual actin filaments as they entered the ring (Fig. 3), as if the actin filament were being actively pulled into the ring and aligned horizontally within the ring. If indeed a pulling force exists, we speculate that a myosin motor, most likely myosin II, is responsible for pulling the actin filament into the ring, after the filament is transported by microtubules to the vicinity of the ring. This idea is consistent with data suggesting that myosin II is present in the ring prior to the arrival of actin filaments [38]–[40].

To examine filament dynamics, we used low-level, Qdot-conjugated phalloidin as a novel means of “speckling” actin in living cytokinetic cells. Qdot-conjugated phalloidin has been used to label actin in vitro [41] and in semi intact cells [42]. It has also been used to label cortical actin in living cells, but not by “speckling” – as multiple dots per filament were not observed [43]. This labeling scheme, in conjunction with Alexa 488 phalloidin label, allowed us to investigate individual actin filaments as they entered into and exited from the dynamic contractile ring. By speckling a phalloidin-labeled filament with Qdot reference points, we were able to determine that intact actin filaments can enter and exit from the ring. In other words, these filaments do not appear to be undergoing rapid polymerization or depolymerization. This finding confirms not only that the ring is not built exclusively via on-site de novo assembly, but also that disassembly of the ring is not accomplished exclusively by depolymerization of filaments within the ring. We have considered the possibility that phalloidin could interfere with the assessment of filament stability by stabilizing the filaments. However, Cao and Wang demonstrated that cells injected with rhodamine phalloidin divided normally even at intracellular concentrations of up to ∼1.65 µM [19], suggesting that minimal stabilization occurred. In our experiments, the intracellular concentration was approximately 0.2 µM, a level at which de novo assembly of actin filaments could occur [17]. Thus, we believe that any stabilization of actin filaments induced by this level of phalloidin is likely to be minimal.

Conjugated-Qdot technology has its pros and cons [44]. Qdot photostability would be an asset in Fluorescence Speckle Microscopy [45], [46] applications and could permit tracking of actin dynamics in processes that depend on actomyosin based contraction. However, Qdots present a technical challenge due to their tendency to aggregate. Aggregation is minimized by suspension in buffer of pH 8.3 and increases with decreasing pH. Because the intracellular pH is below 8.3, the possibility of intracellular aggregation must be considered. For example, it appears as if some Qdots in Video S5 increase in size over time. At the start of the video, the dots are uniformly small, yet by the end, there are a number of large dots. Perhaps as the dots converge on the contractile ring, thus becoming more concentrated, they are more likely to aggregate into clusters. This could create artifacts if multiple actin filaments, linked via aggregated label, travel together towards the ring, perhaps hitching a ride on a single microtubule motor protein. Despite the potential for aggregation, actin filaments could still be seen entering and exiting the ring. However, this does illustrate the importance of minimizing the concentration of injected Qdots.

During late anaphase, a large fraction of the cellular actin is recruited to build a robust contractile ring, ensuring completion of cytokinesis even under unfavorable conditions. Actin filaments arrive from multiple subcellular locations via diverse mechanisms of transport, providing an ample supply of actin for the contractile ring. Our micromanipulation and actin-tracking data indicate that in one mechanism of actin redistribution, filaments are recruited to the furrow region by microtubule-mediated transport. We have also documented the presence of two previously characterized means of redistributing actin into the contractile ring. First, in the de novo assembly mechanism, actin can assemble into filaments at the plus ends of central spindle microtubules and subsequently be transported to the equatorial cortex, as these microtubules splay out laterally towards the cell periphery [17]. Incidentally, vesicle-associated actin in *Drosophila* [47] may also undergo lateral transport. Second, the cortical flow mechanism involves microtubule-mediated flow of actin filaments from the polar cortex towards the equatorial cortex [17], [18]. Notably, all these mechanisms are mediated by microtubules. These new data show that, in grasshopper spermatocytes, actin redistribution by transport along microtubule tracks does not function in isolation. Further, the data promote cross-species comparison. Given the commonality of these two mechanisms in silkworm and grasshopper spermatocytes, it becomes plausible that actin transport along microtubule tracks may also function in silkworm. However, it remains to be determined which if any mechanism predominates.

Other potential pathways may play minor roles in redistribution. For instance, actin filaments might be siphoned out of cell division scars, along microtubules, and recycled into the ring (see Fig. 1C, a–c, Fig. 8D). Spindle-associated mitochondria (which can be transported along, or anchored to, actin filaments [34]) could serve as a “magnet” for attracting actin subunits or filaments to the vicinity of the equatorial cortex. This raises the question of whether some of the staining that flanks the central spindle (Fig. 3) could be due to actin (labeled green) bound to mitochondria, rather than merely green auto-fluorescence from the mitochondria. Again, the contribution of this hypothesized pathway may be minimal, as removal of all detectable mitochondria from the central spindle region (Fig. 9) does not impair furrow induction or ingression. Recent investigations on mouse oocytes and *Xenopus* embryos have revealed intriguing spindle-shaped “cages” composed of actin filaments or cables, which surround and sometimes penetrate the spindle. In addition to positioning the spindle and/or regulating its length [32], [48], perhaps the actin from such structures could ultimately be recycled into the ring by traveling down spindle microtubules.

## Materials and Methods

### Preparation and micromanipulation of grasshopper spermatocytes

Primary cultures of spermatocytes were prepared from the grasshopper *Melanopus femurrubrum*, and the cells were micromanipulated as previously described [17], [27], [49]. In brief, a monolayer of spermatocytes was spread on a coverslip under inert halocarbon oil. To improve optical clarity, all cells were flattened during preparation. Cells were micromanipulated with a fine glass needle (tip diameter ∼0.1 µm) maneuvered using a Burleigh MIS-5000 series piezoelectric micromanipulator.

### Immuno-labeling

Cells were fixed and stained as previously described [27], [50]. In brief, cells were spread on coverslips and prefixed for five minutes by micropipetting a microfixative (2% Glutaraldehyde, 1% Chaps, 0.33 mM Rhodamine phalloidin (Invitrogen) in potassium Pipes buffer) in their vicinity. The cells were then incubated for 10 minutes in macrofixative (0.1% Glutaraldehyde, 0.5% NP-40 in potassium Pipes buffer). Microtubules were stained using a 1∶500 dilution of anti-β tubulin (clone KMX-1, Chemicon) primary antibody and a 1∶100 dilution of Alexa-fluor 488 conjugated goat anti-mouse secondary antibody (Invitrogen). Actin filaments were stained with 0.165 mM Rhodamine phalloidin (Invitrogen). Cells were mounted using Vectashield mounting medium (Vector Laboratories), containing DAPI to stain chromosomes.

### Conjugation of Qdots to Phalloidin

At the time this experiment was performed, preconjugated Quantum Dots were available commercially from Qdot. Qdot technicians conjugated Qdot 655 ITK Amino (PEG) Quantum Dots (Qdot) to Amino Phalloidin, Hydrochloride (Alexis Biochemicals) with the crosslinker *Bis* (sulfosuccinimidyl) suberate (Pierce Chemicals). Qdots are currently available from Invitrogen in their unconjugated form; Invitrogen provides a detailed protocol for conjugation of Qdots to phalloidin on their web site.

### Microinjection

Rhodamine phalloidin, Alexa 488 phalloidin and Qdot 655 phalloidin conjugates were introduced into cells using a custom-made, pressure-controlled microinjection device. Qdot conjugates (1 µM in pH 8.3 borate buffer) tended to aggregate within the microinjection needle, even though pre-existing aggregates were removed by centrifugation immediately before the needle was loaded. To compensate for the aggregation, we used a microinjection needle of diameter approximately 1.5 times larger than that of our standard microinjection needles, which are smaller than 0.1 µM.

### Live Cell Labeling with the Tubulin Tracker

Tubulin Tracker (Invitrogen) was used as directed, with the Oregon Green 488 Taxol (paclitaxel) diluted to 50 µM in insect Ringer's solution. The diluted Tubulin Tracker was micropipetted around the target cells, resulting in a further dilution of at least 1000 fold.

### Microscopy

Microscopy (digital-enhanced polarization, DIC, and fluorescence) was performed with a custom-modified Axiovert 100 inverted microscope (Carl Zeiss, Inc). The microscope was equipped with an Ellis optical fiber light scrambler (Technical Video, Woods Hole, MA) and a Glan-Thompson polarizer. Cells were viewed using an infinity-corrected 1.4 NA/63× Plan-Apochromat objective lens and a 1.4 NA achromatic-aplanatic condenser (Carl Zeiss, Inc). Cells were imaged using cooled-CCD digital cameras: MicroMax (Princeton Instruments); ORCA-100, Model C4742-95 (Hamamatsu); and EM-CCD, Model C9100-12 (Hamamatsu). Images were acquired and processed with Image Pro Plus (Media Cybernetics) and SimplePCI (C-Imaging Systems) software.

Image stacks were acquired using a laser scanning confocal microscope (Leica TCS), processed in Photoshop 5.0 (Adobe), and reconstructed using SimplePCI (C-imaging Systems).

## Supporting Information

Video S1 Mechanically induced reorganization of spindle microtubules generated ectopic furrows. Video S1 corresponds to Figure 1B. The cell was imaged using polarization microscopy. Following anaphase onset (shown in Fig. 1B, 0 min), the spindle was collapsed with a microneedle. Lateral growth of the reorganizing microtubules dislodged mitochondria, driving them toward the distal ends of the microtubule bundles. On opposite sides of the cluster of chromosomes, two microtubule bundles assembled into lateral spindles, while the displaced mitochondria re-bundled with the microtubules. Cleavage furrows initiated simultaneously around both of the continuously reorganizing lateral spindles. Ultimately, each furrow ingressed around the approximate midpoint of the spindles, producing two anucleate membrane pockets. Chromosomes were clearly visible in the interior of the cell throughout the sequence.
(1.17 MB MOV)

Video S2 Furrow repositioning induced by reorganization of a mechanically folded spindle. Video S2 corresponds to Figure 2B. The cell was imaged using polarization microscopy. After the spindle was folded in half along its long axis (prior to start of video), the spindle microtubules reorganized into a single bundle. Microtubules from the joint spindle poles radiated toward the sides of the cell cortex, where a furrow initiated. Furrow ingression was accompanied by a relative realignment between spindle and furrow such that the furrow ultimately ingressed at the spindle equator. Chromosomes were clearly visible in the interior of the cell throughout the sequence.
(0.91 MB MOV)

Video S3 Incorporation of actin filaments into the contractile ring during furrow induction. Video S3 corresponds to Figure 3A. A surface (cortical) view of furrow induction. Alexa 488 phalloidin-labeled actin filaments were imaged every 10 seconds. Just prior to furrow initiation, some cortical actin filaments began to move toward the spindle equator where they coalesced into nascent bundles within the assembling contractile ring. The arrow tracks the movement of an actin filament or cable as it moved toward the equator, changed its trajectory, and disappeared into the ring. Note the steady increase in fluorescence at the nascent ring due to incorporation of fluorescent actin filaments.
(5.87 MB AVI)

Video S4 Incorporation of actin filaments into the contractile ring during furrow ingression. Video S4 corresponds to Figure 3B. A midplane view of furrow ingression. Alexa 488 phalloidin-labeled actin filaments were imaged every 10 seconds. During furrow ingression, actin filaments continued to move toward and incorporate into the contractile ring. The arrow tracks an actin filament or cable as it moved into the ingressing contractile ring.
(1.91 MB AVI)

Video S5 Redistribution of actin filaments labeled by Qdot 655-Phalloidin. Video S5 corresponds to Figure 4. Actin filaments were labeled by microinjection of phalloidin-conjugated Qdot 655 into a spermatocyte. At the onset of cytokinesis, the Qdot-decorated actin filaments formed a barely distinct pattern, vaguely reminiscent of the distribution of central spindle microtubules. (Note that the spindle-like pattern was unavoidably superimposed on a cortical flow-derived pattern, as the Qdots can label cortical actin filaments as well as cytoplasmic filaments.) Qdots gradually accumulated at the cleavage furrow as cytokinesis proceeded. They were largely excluded from the region of the segregated chromosomes (dark spot in the center of each nascent daughter cell).
(9.92 MB AVI)

Video S6 Redistribution of actin filaments labeled by Alexa 488-Phalloidin and Qdot 655-Phalloidin during furrow induction. Video S6 corresponds to Figure 5A. Alexa 488-Phalloidin labeled actin filaments (green), speckled with Qdots (red), continuously merged into the contractile ring during furrow induction. The unlabeled spindle was flanked by a pair of green bundles that contained microtubule-associated, autofluorescent mitochondria.
(10.23 MB AVI)

Video S7 Redistribution of actin filaments labeled by Alexa 488-Phalloidin and Qdot 655-Phalloidin during furrow ingression. Video S7 corresponds to Figure 5B. Alexa 488-Phalloidin labeled actin filaments (green), speckled with Qdots (red), continuously merged into the contractile ring during furrow ingression, as seen in this midplane view. Occasionally, Qdot-decorated actin filaments could be seen moving away from the contractile ring. Despite the short timeframe of the video, there was noticeable clearing of actin from the non-equatorial region. Videos S8, S9, S10 highlight three regions of interest within the cell shown in Video S7.
(10.21 MB AVI)

Video S8 Entry into the ring of a labeled actin filament speckled with Qdot 655-Phalloidin. Video S8 corresponds to Figure 6A. Cells were colabeled by microinjection with Alexa 488 phalloidin and Qdot 655 phalloidin conjugates. This protocol generated green phalloidin-stained actin filaments that were marked with red photo-stable speckles. The arrow tracks two Qdots on an actin filament or cable as the actin moved toward the cleavage furrow (left side).
(0.07 MB AVI)

Video S9 Entry into the ring of a labeled actin filament speckled with Qdot 655-Phalloidin. Video S9 corresponds to Figure 6B. Cells were colabeled by microinjection with Alexa 488 phalloidin and Qdot 655 phalloidin conjugates. This protocol generated green phalloidin-stained actin filaments that were marked with red photo-stable speckles. The arrow tracks two Qdots on an actin filament or cable as the actin moved toward the cleavage furrow (left side).
(0.10 MB AVI)

Video S10 Exit from the ring of a labeled actin filament speckled with Qdot 655-Phalloidin. Video S10 corresponds to Figure 6C. Cells were colabeled by microinjection with Alexa 488 phalloidin and Qdot 655 phalloidin conjugates. This protocol generated green phalloidin-stained actin filaments that were marked with red photo-stable speckles. The arrow tracks two Qdots on an actin filament or cable as the actin moved away from the constricting cleavage furrow (left side).
(0.09 MB AVI)

Video S11 Cortical flow of actin filaments in grasshopper spermatocytes. Video S11 corresponds to Figure 8A. Time-lapse sequences of fluorescence images, captured every 30 seconds, showed cortical flow of actin filaments labeled with Alexa 488 phalloidin. Flow of actin was induced by collapsing the spindle (labeled “sp” in Figure 8A) and repositioning it near the cortex. Note that the signal cleared over time in the region closest to the spindle, as the actin flowed to the cortex on the opposite side of the cell. The displaced, autofluorescent mitochondria were seen as large white patches within the cell (labeled “m” in Fig 8A). The mitochondria were not part of the cortical flow; rather their displacement was presumably due to the elongation of dynamic spindle microtubules, with which they were associated.
(3.35 MB AVI)
